# Provider Perspectives on the Role of Hypotension in Pediatric Kidney Transplant: A Pediatric Nephrology Research Consortium Study

**DOI:** 10.1111/petr.70130

**Published:** 2025-07-18

**Authors:** Hailey Connolly, Olga Charnaya, Sruthi Tatavarthi, Shireen Hashmat

**Affiliations:** ^1^ Department of Pediatrics Hackensack University Medical Center Hackensack New Jersey USA; ^2^ Department of Pediatrics Johns Hopkins University School of Medicine Baltimore Maryland USA; ^3^ Johns Hopkins University Baltimore Maryland USA; ^4^ Department of Pediatrics University of Chicago Chicago Illinois USA

**Keywords:** dialysis, hypotension, pediatric kidney transplant

## Abstract

**Background:**

The lack of a consistent definition for hypotension in the pediatric population complicates the study of hypotension in pediatric kidney transplant recipients. Additionally, there is limited data to guide the management of hypotension in these patients both before and after transplantation.

**Methods:**

A 10‐question survey was distributed to pediatric nephrologists. The survey data was analyzed using descriptive statistical methods without hypothesis testing.

**Results:**

A total of 25 respondents completed the survey, revealing significant variability in the definitions of hypotension used and in the management approaches for hypotension during the pre‐ and posttransplant periods.

**Conclusions:**

There is significant variability in practice patterns regarding the management of hypotension in pediatric transplant recipients. This highlights the need for further research to evaluate the prevalence of chronic hypotension in the pediatric ESKD population and to assess its impact on transplant outcomes.

**Abbreviations**
ESKD: end‐stage kidney disease
PNRC: Pediatric Nephrology Research Consortium

## 1 Introduction

Pediatric kidney transplant recipients face unique clinical challenges compared to adult kidney transplant recipients stemming from differing etiologies of end‐stage kidney disease (ESKD), a notable size mismatch between donor and recipient, and differing cardiovascular risk profiles [1]. Persistent pretransplant hypotension has anecdotally been considered a relative contraindication to transplant due to concern for allograft thrombosis and non‐function due to hypoperfusion [2]. The field has, however, been hampered from studying this due to inconsistent definitions of hypotension, differing clinical practices impacting access to transplantation, and extremely limited data on posttransplant outcomes of pediatric ESKD patients with hypotension [3, 4].

Due to the lack of a consistent and unified definition of hypotension, combined with the rarity of pediatric ESKD patients experiencing significant hypotension, there have been very few studies evaluating the impact of hypotension on kidney transplantation outcomes. Several guidelines address hypotension only as a side effect of other therapies and not as the primary hindrance to transplant function [5, 6]. The Pediatric Continuous Renal Replacement Therapy Workgroup has defined intradialytic hypotension as the combination of systolic blood pressure < 5th percentile for age and/or clinical symptoms; however, they do not address persistent hypotension outside of the dialysis setting [7]. Management of persistent and refractory hypotension in pediatric patients with ESKD is limited to descriptions found in case reports, case series, and clinician experiences [8, 9, 10].

The aim of our study is to describe the clinical definition of pretransplant hypotension and identify the common etiologies and treatment modalities used for the management of pre‐ and posttransplant hypotension derived from surveys of practicing pediatric nephrologists.

## 2 Methods

The survey [Data S1] used for this study was approved by the Clinical Research Review Committee of the Pediatric Nephrology Research Consortium. The survey was shared at the PNRC Spring 2024 meeting, through the American Society of Nephrology Pediatric Community of Practice, and the Pediatric Nephrology Listserv. Survey data was analyzed using descriptive statistical methods without hypothesis testing. Analysis was performed using STATA version 17. This study was determined exempt from IRB by the University of Chicago's IRB (IRB23‐2090).

## 3 Results

**FIGURE 1:**
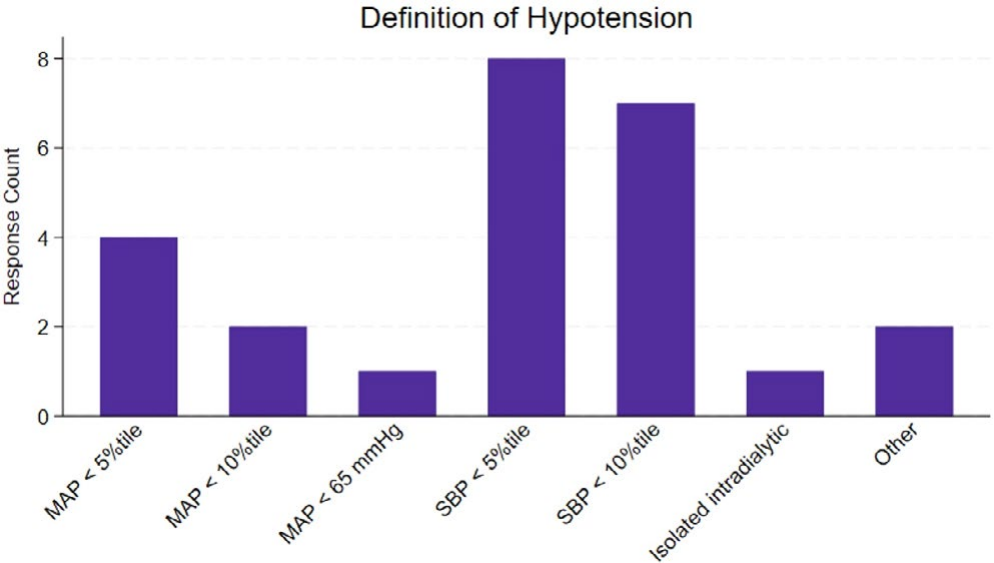
Distribution of preferred definition for sustained hypotension. The most common definitions are systolic blood pressure (SBP) below the 5th percentile, with eight responses (32%), and SBP below the 10th percentile, with seven responses (28%). Mean arterial pressure (MAP) less than the 5th percentile is the third most frequently cited definition, with four responses (16%). Fewer respondents defined hypotension as MAP below the 10th percentile, MAP below 65 mmHg, or isolated intradialytic hypotension, each receiving 1–2 responses.

There was a total of 25 respondents to the survey, all of whom identified as pediatric nephrologists. As illustrated in Figure 1, the most commonly used definitions for hypotension among respondents were systolic blood pressure less than the 5th percentile (*n* = 8, 32%) or systolic blood pressure less than the 10th percentile (*n* = 7, 28%). Frequently observed etiologies of hypotension included vasoplegia, anephric status, dialysis‐associated hypotension, and cardiac pathology.

**FIGURE 2:**
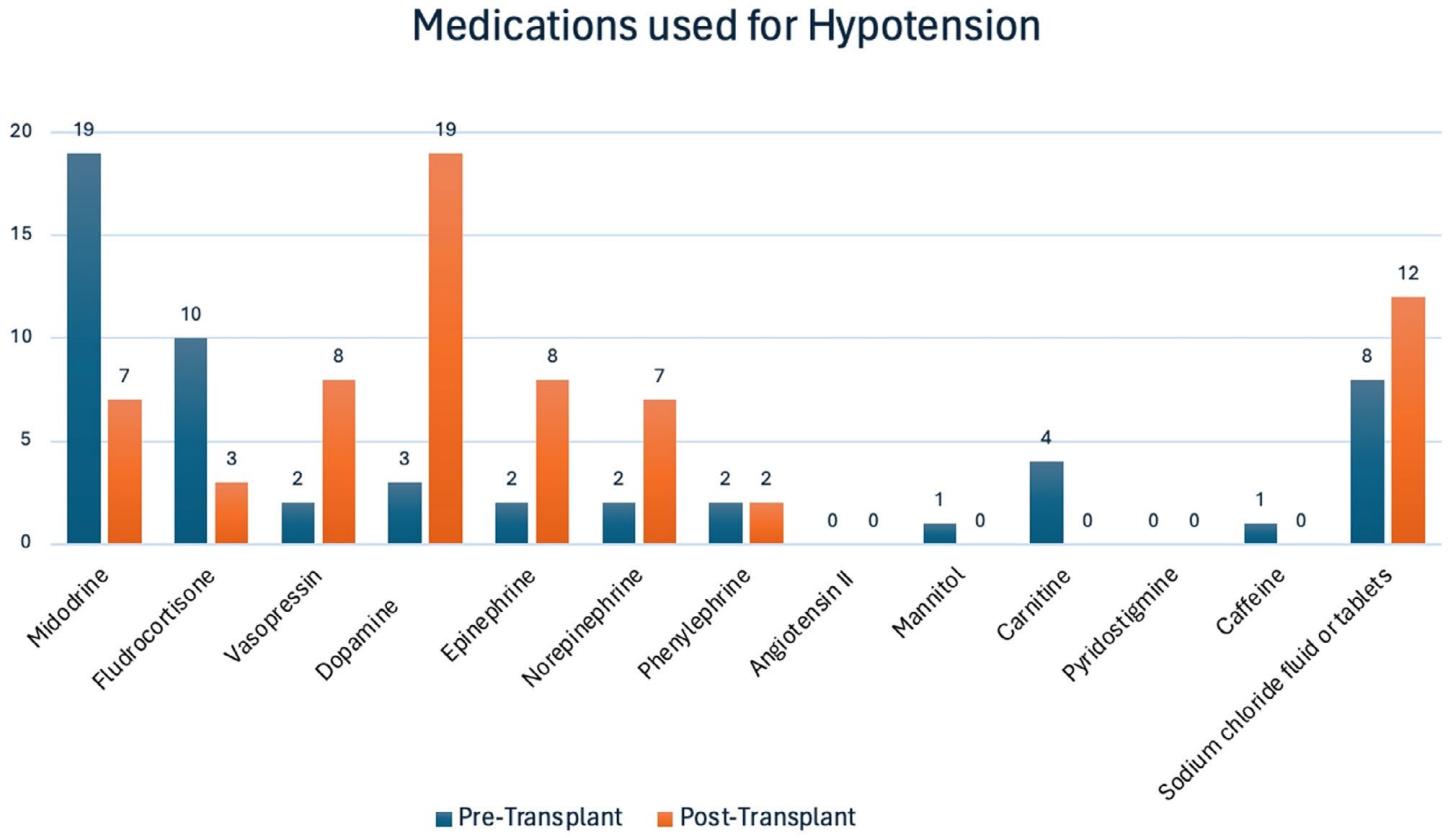
Distribution of therapies utilized for hypotension. Respondents were asked to select all medications that they used in their practice. In the pretransplant phase, Midodrine and Fludrocortisone were the most commonly used therapies, with 19 and 10 respondents (76% and 40%), respectively, while dopamine and vasopressin were less frequently utilized. Posttransplant, dopamine and epinephrine emerged as the most used therapies, with 19 and 8 respondents respectively, indicating a shift in medication preferences following the transplant.

Peritoneal dialysis was the preferred dialysis modality in patients with pretransplant hypotension for the majority of pediatric nephrologists (*n* = 23, 92%) as shown in Table 1. Nineteen respondents (*n* = 19, 76%) reported previously referring a patient with hypotension for transplant listing. Only nine pediatric nephrologists surveyed (*n* = 9, 36%) had removed a patient from transplant listing due to hypotension. The duration of sustained hypotension leading to delisting varied from 1 to greater than 4 months. Most respondents (*n* = 19, 76%) had provided care to a patient requiring pharmacologic intervention for hypotension prior to transplant (Table 2). Midodrine, fludrocortisone, and sodium chloride supplements were the most frequently used medications for management of pretransplant hypotension (Figure 2).

**TABLE 1:** Characteristics of preferred modalities for patients with sustained hypotension.

Dialysis modality	Frequency of pediatric nephrologists
Peritoneal dialysis	23
Continuous renal replacement therapy	2
Hemodialysis	0

*Note:* Ninety two percent of respondents (*n* = 23) favor peritoneal dialysis as the modality of choice, while only two respondents (8%) use Continuous Renal Replacement Therapy (CRRT). This data highlights the predominant reliance on Peritoneal Dialysis in pediatric nephrology practice for hypotensive patients.

**TABLE 2:** Utilization of Vasoactive Medications for Hypotension Management Prior to Transplant in Patients.

Patients requiring vasoactive medications for hypotension prior to transplant	Frequency of pediatric nephrologists
No	6
Yes	19

*Note:* Seventy six percent of pediatric nephrologists (*n* = 19, 76%) reported that their patients required pharmacological intervention for hypotension prior to transplant. Only six nephrologists (24%) reported that their patients did not require these medications before the transplant.

In the first 24–48 h following renal transplant, pediatric nephrologists reported different blood pressure goals, with the most frequently cited target for the lower limits of systolic blood pressure (SBP) being greater than the 50th percentile. Medications commonly used to manage posttransplant hypotension included dopamine and sodium chloride, in the form of intravenous fluids or tablets; however, their use varied significantly among respondents between the pre‐ and posttransplant periods. Among those who had cared for a patient with pretransplant hypotension, it was identified as the cause of allograft failure in eight patient cases.

## 4 Discussion

This survey provides insights into the practices and perspectives of pediatric nephrologists regarding the definition, management, and outcomes of pretransplant hypotension. Overall, the survey results reinforced the lack of a consistent definition for hypotension among pediatric nephrologists, with 60% of respondents using either systolic blood pressure less than the 5th or 10th percentile. This variability likely reflects the lack of a standardized definition for pediatric hypotension.

The reported etiologies of hypotension, including notably vasoplegia, anephric status, dialysis‐associated hypotension, and cardiac pathology, are consistent with previously published pediatric data. The majority of survey respondents chose peritoneal dialysis (92%) as a preferred modality in managing pediatric patients with pretransplant hypotension. This preference likely results from less hemodynamic instability with peritoneal dialysis compared to hemodialysis. Eight percent of respondents reported use of CRRT as the preferred dialysis modality in ESKD patients with hypotension. These patients possibly remained inpatient prior to transplantation.

Pretransplant hypotension was frequently (76%) treated with medications such as midodrine, fludrocortisone, and sodium chloride supplements, being the most used agents, highlighting the variability of medical management among pediatric nephrologists. Although our survey reflects a small sample of the pediatric nephrology provider's community, the majority of respondents (76%) have referred a patient with hypotension for transplant listing, and only 36% of respondents had delisted a patient due to sustained hypotension, suggesting that many pediatric nephrologists anticipate successful outcomes and resolution of hypotension post kidney transplant.

Posttransplant blood pressure management goals varied among the respondents, with the lower limit of systolic blood pressure often targeted to be greater than the 50th percentile. This target reflects the importance of maintaining adequate perfusion to the transplanted kidney. It is noteworthy that a wide range of agents was used, with dopamine and sodium chloride (either as intravenous fluids or tablets) being most common. This variation likely stems from differences in patient‐specific factors, institutional protocols, and provider preferences. Additionally, hypotension was identified as the cause of allograft failure in eight responses, indicating a need for rigorous pre‐ and posttransplant blood pressure management strategies.

Limitations of this study include a small sample size as well as lack of representation from transplant surgeons. Two respondents reported that the lower limit SBP goal in the first 24–48 h posttransplant is determined based on the transplant surgeon recommendation. It would therefore be informative to include analysis of surgeon practice preferences in future studies. In an upcoming retrospective study, we seek to further evaluate the scope of chronic hypotension in the pediatric nephrology population and determine the impact of pretransplant hypotension on delayed graft function (DGF), allograft function over time, and patient survival.

In conclusion, this survey highlights the variability in the definition, management, and outcomes of pretransplant hypotension among pediatric nephrologists. There is a clear need for standardized definitions and guidelines to enhance consistency in clinical practice.

## Supporting information


Data S1.

## Data Availability

The data that support the findings of this study are available on request from the corresponding author. The data are not publicly available due to privacy or ethical restrictions.
