# Supplementary material for: Provider Perspectives on the Role of Hypotension in Pediatric Kidney Transplant: A Pediatric Nephrology Research Consortium Study
Source: Pediatr Transplant. 2025 Jul 18;29(5):e70130. doi: 10.1111/petr.70130 (PMC12274291; doi:10.1111/petr.70130)
Supplement: Supplementary file 1 — Data S1. [file PETR-29-e70130-s001.docx]

Supplementary Material:

Survey Questions

1. What is the definition of hypotension you use in your clinical practice?
   1. Systolic blood pressure < 5th percentile
   2. Systolic blood pressure < 10th percentile
   3. Mean arterial pressure (MAP) < 70
   4. Mean arterial pressure (MAP) < 65
   5. Isolated intradialytic hypotension
2. What etiologies for pre-transplant hypotension have you observed in your patients? (choose all that apply)
   1. Vasoplegia
   2. Anephric
   3. Cardiac etiology: Congenital heart disease/arrhythmias
   4. Postural orthostatic tachycardia syndrome (POTS)
   5. Secondary to dialysis modality
   6. Congenital adrenal hyperplasia (CAH)
   7. Hypothyroidism
   8. Nephrotic syndrome
   9. Other
3. What dialysis modality do you prefer for patients with sustained hypotension in a non-ICU setting?
   1. Peritoneal dialysis
   2. Hemodialysis
   3. CRRT
4. Have you previously referred a patient with hypotension for transplant listing?
   1. Yes
   2. No
5. Have you ever removed a patient from listing (or made inactive indefinitely) due to hypotension?
   1. Yes
   2. No
6. After what duration of sustained hypotension, would you consider removing a patient from transplant listing?
   1. <1 month
   2. 1-2 months
   3. 2-3 months
   4. 3-4 months
   5. >4 months
7. Have you cared for a patient who required vasoactive medications for treatment of hypotension prior to transplant?
   1. Yes- if so, which medications have you used for management of hypotension?
      - Midodrine
      - Fludrocortisone
      - Vasopressin
      - Dopamine
      - Epinephrine
      - Norepinephrine
      - Phenylephrine
      - Angiotensin II
      - Mannitol
      - Carnitine
      - Pyridostigmine
      - Caffeine
      - Sodium chloride fluid or tablets
      - None of the above
      - Other
   2. No
8. What is your goal for the lower limit of the systolic blood pressure(SBP) in the first 24-48 hours post-transplant period?
   1. SBP >5th percentile for age/height
   2. SBP >10th percentile for age/height
   3. SBP >50th percentile for age
   4. Mean arterial pressure (MAP) >60
   5. Mean arterial pressure (MAP) >65
   6. Mean arterial pressure (MAP) >70
9. What pharmacological interventions have you previously used for treatment of hypotension in the first 24-48 hours post-transplant?

- Midodrine
- Fludrocortisone
- Vasopressin
- Dopamine
- Epinephrine
- Norepinephrine
- Phenylephrine
- Angiotensin II
- Mannitol
- Carnitine
- Pyridostigmine
- Caffeine
- Sodium chloride fluid or tablets
- Other

1. If your patient with pre-transplant hypotension experienced allograft failure, what was the attributed etiology?

- Hypotension/hypo-perfusion
- Thrombosis
- Rejection
- Secondary to other organ failure
- Other
